# Characterization of Hapten–Protein Conjugates: Antibody Generation and Immunoassay Development for Pesticides Monitoring

**DOI:** 10.1007/s12668-013-0083-8

**Published:** 2013-03-28

**Authors:** Kumar Rajesh, K. Vikas Rana, C. Raman Suri

**Affiliations:** 1 Department of Biotechnology, Shoolini Institute of Life Science, Solan, Himachal Pradesh India; 2 Institute of Microbial Technology (CSIR), Sector 39-A, Chandigarh, 160 036 India; 3 Present Address: Biomaxe India 905/Industrial area phase II, Chandigarh, India

**Keywords:** Characterization, Hapten–protein conjugates, Fluorescence, Antibodies, Pesticides

## Abstract

The generation of specific and sensitive antibodies against small molecules is greatly dependent upon the characteristics of the hapten–protein conjugates. In the present study, we report a new fluorescence-based method for the characterization of hapten–protein conjugates. The method is based on an effect promoted by hapten–protein conjugation density upon the fluorescence intensity of the intrinsic tryptophan chromophore molecules of the protein. The proposed methodology is applied to quantify the hapten–protein conjugation density of two different class of pesticides (atrazine and 2,4-dichlorophenoxyacetic acid in this study) coupled to carrier protein. The study proved useful for monitoring the course of hapten–protein conjugation for the production of specific antibodies against small molecules. Well-characterized hapten–protein conjugates enabled obtaining highly sensitive anti-atrazine and anti-2,4-D antibodies with IC_50_ values equal to 12 and 70 ng mL^−1^ for atrazine and 2,4-D respectively. These antibodies were used for developing a fluorescence-based immunoassays format demonstrating a detection limit of atrazine and 2,4-D in standard water samples 2 and 7 ng mL^−1^, respectively. The developed immunoassay format could be used as convenient quantitative tools for sensitive and specific screening of pesticides in samples.

## Introduction

Small molecules such as pesticides, drugs, etc. are usually non-immunogenic, and hence do not elicit an immune response unless coupled with some macromolecules such as proteins. It is, therefore, required to modify these small substances (hapten) for coupling with macromolecules (carrier) so as to make a stable carrier–hapten complex. The hapten density of the conjugate is an important parameter which generally defines the quality and quantity of antibody produced against the target molecules. There has been significant progress in recent years in protein–hapten conjugates for the generation of anti-hapten antibodies with applications in immunoassays for environmentally hazardous pollutants [1–3]. An improved method for the micro-scale preparation and characterization of hapten–protein conjugates for nonchromophore hydroxylated haptens was demonstrated by Naar and co-workers [4]. In another study, a solid-phase conjugation method utilizing carrier protein bound to an ion exchange matrix was developed and the conjugation ratio was determined by acid hydrolysis and amino acid analysis [5]. Verification of the coupling reaction and determination of the hapten density can be accomplished spectrophotometerically, mainly by evaluating the available free amino groups before and after conjugation [6, 7], by matrix-assisted laser desorption ionization mass spectrometry (MALDI-MS) to determine the mass change before and after conjugation [8, 9] or by radiolabelled haptens [10]. In order to prepare an effective hapten–protein conjugate for the desired immune response, it is important to characterize the resulting hapten–protein conjugates to determine the hapten density on carrier protein. The higher ratio of hapten usually increases the strength and specificity of the immune response. However, there is a risk that a high degree of substitution could adversely affect the activity and specificity of antibodies produced [11]. Careful selection of hapten–protein conjugates with optimum hapten density is therefore an important aspect for the generation of specific and sensitive antibodies against small molecules.

In the present study, two different haptens, i.e., mercaptopropionic acid derivative of atrazine (MPAD) and activated 2,4-dichlorophenoxy acetic acid (2,4-D), were used to make hapten–protein conjugates, and the binding efficiency of hapten–protein conjugate (number of hapten molecules per protein molecule) was determined fluorometrically and further confirmed by MALDI-TOF mass spectrometer by taking different hapten/protein molar ratios. The specific anti-atrazine antibodies were generated by selecting the conjugate of optimum hapten density for immunization purpose.

## Materials and Methods

Bovine serum albumin (BSA), dicyclohexylcarbodiimide (DCC), *N*-hydroxysuccinimide (NHS) ester, 3-mercaptopropionic acid, peroxidase conjugated goat anti-rabbit immunoglobulin, normal anti-rabbit immunoglobulin, Freund’s complete (CFA) and incomplete adjuvants (IFA) were purchased from Sigma Chemical Co., USA. Technical grade atrazine and 2,4-dichlorophenoxy acetic acid (2,4-D) were purchased from Supelco, USA. Peroxidase (POD) and 3,3′,5,5′-tetramethylbenzidine (TMB) substrate were obtained from Bangalore Genei, India. All chemicals, reagents, and solvents used in this study were of high purity analytical grade. Buffers were made in Milli-Q double distilled water.

### Conjugation of Haptens with Protein

MPAD was synthesized as described earlier [12]. Conjugation of atrazine (MPAD derivative) and 2,4-D with protein (BSA) were made at five different ratio of protein–hapten (1:5, 1:10, 1:20, 1:40, and 1:100). For atrazine conjugates, the protein solution (10 mg/ml; 0.15 μmol) was made in pH 9.0 borate buffer and the final reaction volume of the protein–hapten conjugates was kept constant at 1 ml for each preparation. Stock solution of haptens was made by adding 50 μmol MPAD derivative of atrazine in 1 ml DMF along with 125 μmol of DCC and 125 μmol of NHS. The mixture was incubated for 4 h at RT and then centrifuged for 5 min at 10,000 rpm to remove the urea precipitate. Similarly, the carboxyl groups of 2,4-dichlorophenoxy acetic acid (2,4-D) were activated by carbodiimide activation method using DCC. This was done by dissolving 10 mg of 2,4-D in 1.3 ml dioxane along with 17 mg NHS and 62 mg DCC. The mixture was incubated for 18 h at RT and then centrifuged for 5 min at 10,000 rpm to remove the urea precipitate. The supernatant of activated MPAD and 2,4-D solutions were used to prepare the conjugates with protein by adding different amounts of haptens to a fixed amount of protein (10 mg) in a final volume of 1 ml to make protein–hapten conjugates of different molar ratios (D1-D5 and M1-M5 for 2,4-D and atrazine, respectively) as shown in Table 1. The conjugates were passed through P10 gel filtration column (Pharmacia, Sweden). The fractions with maximum protein concentration were collected, pooled, and checked for the final concentration of protein (hapten–protein) with UV spectrophotometer at 280 nm.

**Table 1:** Molar ratios used for making MPAD-protein (M1–M5) and 2,4-D-protein conjugates (D1–D5)

Protein concentration, μmol (10 mg)	Hapten concentration	Protein–hapten ratio
0.15	0.75 (15 μl)	1:5 (M1 and D1)
0.15	1.50 (30 μl)	1:10 (M2 and D2)
0.15	3.00 (60 μl)	1:20 (M3 and D3)
0.15	6.00 (120 μl)	1:40 (M4 and D4)
0.15	15.0 (300 μl)	1:100 (M5 and D5)

### Characterization of Conjugates

#### Fluorescence Spectra

The emission spectra of hapten–protein conjugates (D1 to D5 and M1 to M5) were taken in the range of 300–450 nm after excitation at 290 nm (tryptophan excitation) using Kontron spectrofluorometer (SFM 25). The conjugates were also excited at 270 nm to see the effects of both tyrosine and tryptophan amino acids on the conjugates. Spectra were obtained using 1-nm excitation and emission slits, and were recorded at a scan rate of 5 nm s^−1^.

#### MALDI-TOF

The mass analysis of hapten–protein conjugates was done with MALDI spectrometer (Kratos Analytical Systems). Sinapinic acid solution was prepared at a concentration of 15 mg/ml in acetonitrile. Protein conjugates dialyzed against distilled water were used at a concentration of 1.5 mg/ml. The conjugate samples and matrix solution were mixed in equal amounts (1 μL each) and added on the stainless steel probe having 0.5 μL of TFA solution (0.1 %). The samples were allowed to dry at room temperature, and then kept in the system for mass analysis. The data were acquired with 50 shots per sample in the linear mode at 30 kV and analyzed using the software provided with the system.

## Antibodies Production and Purification

The New Zealand white rabbits were immunized with hapten (2,4-D or MPAD) conjugated to carrier protein (KLH or BSA). Rabbits were injected subcutaneously with 300–400 μg of conjugate emulsified in CFA for first injection and in IFA for booster doses which were repeated three to four times every 21 days and sera were collected on fifth day after each booster. After de-complementation at 56 °C for 30 min, it was stored in aliquots at −20 °C. The sera were pooled and precipitated under constant stirring at 4 °C at 50 % saturated ammonium sulfate and centrifuged. The precipitate was dissolved in minimum volume of PBS and was extensively dialyzed against PBS (pH 8.0) at 4 °C. It was then passed through the protein A sepharose column and bound antibody was eluted with 0.1 M glycine-HCl buffer (pH 2.5). Fractions were neutralized immediately with 1 M Tris (pH 8.0) and dialyzed extensively against PBS pH 7.4 at 4 °C, and stored in aliquotes at –20 °C. Antibodies were further purified by passing through BSA–sepharose column to remove anti-BSA antibodies, and its concentration was determined by taking absorbance at 280 nm and by taking 1.35 as extinction coefficient.

## Antibodies Reactivity on Conjugated Hapten-Coated Microtiter Plates

The microtitre plates were coated with protein–hapten conjugates (5 μg mL^−1^) and incubated O/N at 4 °C. After washing with PBS, the non-specific sites were blocked by incubating with 10 % skim milk (in PBS) for 2 h at 37 °C. The test sera/purified antibody were added (100 μl/well) and incubated at 37 °C for 2 h. After washing, the plates were incubated with goat anti-rabbit IgG-HRP (1:5,000 dilution in PBS containing 0.1 % skim milk) for 1 h at 37 °C followed by the addition of TMB substrate for color development. O.D. was measured at 450 nm using ELISA reader (Molecular Devices, USA).

### Competitive Inhibition Fluorescence Immunoassay

ELISA plates were coated with both hapten and conjugated haptens (2,4-D-BSA and MPAD-BSA). The plates were incubated with antibody (1 μg mL^−1^), pretreated with different concentrations of free haptens (5 ppm to 5 ppt made in distilled water) for 2 h at 37 °C. After washing, the plates were incubated with goat anti-rabbit IgG-FITC (1:100 dilution) in PBS containing 0.1 % skim milk for 1 h at 37 °C followed by fluorescence intensity measurement on multimode micrititer plate reader. Data analysis of competitive inhibition assay was performed by normalizing the absorbance using the following formula:

$$ \%\ B/{B_0} = \left( {F - {F_{\mathrm{ex}}}} \right)/\left( {{F_0} - {F_{\mathrm{ex}}}} \right) \times 100 $$

where *F*, fluorescence intensity of hapten at standard concentration; *F*_0_, fluorescence at zero hapten; and *F*_ex_, fluorescence at excess of hapten.

Cross-reactivity of related analoges were calculated on the basis of standard calibration curves in a range of nanogram per milliliter to subnanogram per milliliter level. The data were normalized by %*B*/*B*_0_ transformation and the specific hapten concentration yielding 50 % inhibition used to calculate the cross-reactivity according to the formula:

$$ \%\ \mathrm{Cross}\ \mathrm{reactivity} = H/C \times 100 $$

Where, *H* is the concentration of standard hapten at 50 % *B*/*B*_0_ and *C* is the concentration of cross reacting hapten/analog at 50 % *B*/*B*_0_

## Results and Discussion

The generation of specific and sensitive antibodies against small molecules such as drugs, pesticides, etc. is greatly dependent upon the characteristics of the hapten–protein conjugates. These molecules are synthesized and conjugated with carrier proteins in such a way that they mimic the structure of the compound and contain a reactive group that can form a covalent linkage with the carrier proteins. The functional group of the hapten governs the selection of the conjugation method to be employed. The method used in this study utilized carbodiimide method [13] for carboxy acid haptens to link with BSA. This approach ensured stable cross-linking of haptens with protein along with *N*-acylurea formation. The hapten–protein conjugates of different molar ratios (D1–D5 and M1–M5) were made as shown in Table 1.

**Fig. 1:**
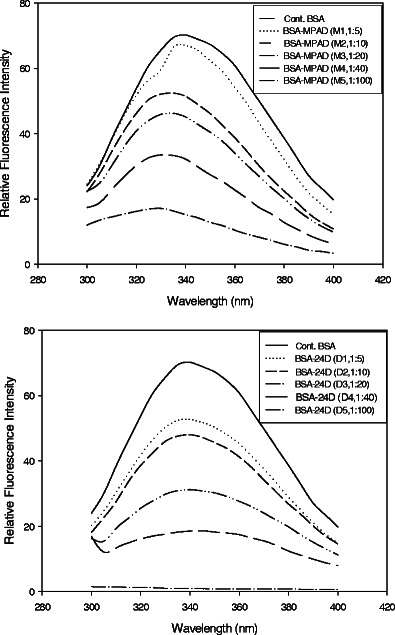
Relative fluorescence intensity spectra for BSA-MPAD and BSA-2,4-D conjugates with change in degree of hapten substitution per protein

**Fig. 2:**
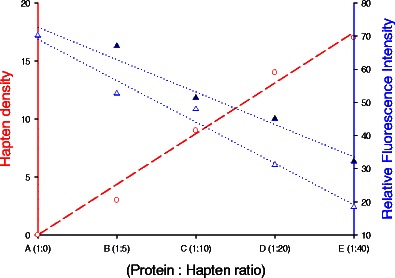
The correlation between hapten–protein conjugation density as determined by MALDI-TOF for different ratio of hapten–protein conjugates (*circle with dashed line*) and the relative fluorescence intensity for conjugates: **a** 2,4-D-protein conjugate (*solid triangle with dotted line*) and **b** atrazine–protein conjugate (*empty triangle with dotted line*)

The fluorescence emission spectra for each hapten–protein conjugate in the range of 300–450 nm after excitation at 290 nm (excitation of tryptophan residues only) are shown for 2,4-D and atrazine, respectively (Fig. 1a, b). Conjugates with higher molar ratios of protein–hapten showed linear decrease in fluorescence intensity. The fluorescence signal at 290-nm excitation and 340-nm emission is mainly due to a number of tryptophan residues being available in the protein. The decrease in fluorescence intensity at this wavelength is due to quenching of tryptophan intensity with increase in hapten density, i.e., increase in the number of amide bonds formed between the surface lysine groups of the protein and carboxylated hapten. This gradual shift in the fluorescence signal or quenching of tryptophan intensity with different conjugates thus confirms the course of hapten–protein conjugation. The number of hapten molecules linked to protein was further confirmed by analyzing mass spectrum obtained by MALDI-TOF spectrometer. Conjugation density for each hapten in increasing molar ratio resulted in a detectable increase in the molecular weight of the conjugate as determined by observing the peak shift of mass spectrum for 2,4-D-protein and atrazine–protein conjugates (Table 2). The molecular weight of each conjugate was calculated from the peak centroid of the peaks. BSA molecule with molecular weight of 66,531 Da (from the manufacturer’s data sheet) showed a gradual shift in mass peaks with conjugates made with different molar ratios of haptens. The incremental change in molecular weight due to incorporation of hapten molecules to protein corresponds to number of hapten molecules per protein molecule. Figure 2 shows the effect promoted by hapten–protein conjugation density as determined by MALDI-TOF upon the fluorescence intensity of the intrinsic tryptophan chromophore molecules. The curve shows the linear relationship between hapten density as determined by MALDI-TOF and relative fluorescence intensity. A standard protein–hapten conjugate made by reacting hapten and protein in a ratio 1:30 was fitted in the standard curve and corresponding fluorescence intensity was measured. The hapten density of the conjugate was determined by correlating with the relative fluorescence intensity from the standard curve (*r* = 0.92).

**Table 2:** Determination of hapten density on BSA-MPAD conjugates using MALDI-TOF mass spectrometric methodThe number of lysine groups on protein being utilized for conjugates before and after conjugation are given in brackets for (a) 2, 4-D-protein conjugates and (b) atrazine–protein conjugates

2, 4-D-protein conjugates				
Sample	BSA-2,4D conjugate	Observed mass^a^ (kD)	Change in mass (∆M) in dalton	Approximate no. of 2,4-D molecules/BSA molecule^b^
D0	BSA	67,221.0		0
D1	1:5	67,962.6		3.35 (3)
D2	1:10	69,171.5		8.82 (9)
D3	1:20	70,228.0		13.60 (14)
D4	1:40	70,926.8		16.76 (17)
D5	1:100	Did not fly	–	–
Atrazine–protein conjugates				
Sample	BSA-MPAD conjugate	Observed mass^a^	Change in mass (∆M) in dalton	Approximate no. of 2,4-D molecules/BSA molecule^b^
M0	BSA only	67,221.0	0	0
M1	1:5	67,744.9	523.9	1.84 (2)
M2	1:10	68,293.8	1,072.8	3.76 (4)
M3	1:20	70,288.7	3,067.7	10.75 (11)
M4	1:40	71,720.8	4,499.8	15.77 (16)
M5	1:100	Did not fly	–	–

^a^Mass calculated from the average of three scans. Each scan was averaged from 50 profiles

^b^Number of haptens molecules per molecule of BSA were calculated by determining the difference in molecular weight (∆M)/molecular weight of hapten

The hapten density of the conjugate is an important parameter which generally defines the quality and quantity of antibody produced. Table 3 shows the pattern of reactivities (IC_50_) and detection limit (LD_10_) values for anti-atrazine antibodies generated against all five conjugates (M1 to M5). It has been observed that the higher antibody titer with moderate antibody affinities is obtained with hapten density of 15–30 molecules per carrier protein (for M3 conjugate). Similar observations have also been reported by other groups [14, 15] where it has been shown that the lower hapten density induces a slower immune response while higher substitution resulted in an IgM response exceeding that of IgG and produced antibodies of lower affinity.

**Table 3:** IC_50_ and LD_10_ values for anti-atrazine antibodies generated against MPAD-protein conjugates (M1 to M5)

Sample no.	Antibodies	IC_50_ (ppb)	LD (10 %) (ppb)
1	RαMPAD (M1)	30.237	0.75
2	RαMPAD (M2)	46.055	0.23
3	RαMPAD (M3)	36.455	0.57
4	RαMPAD (M4)	77.025	0.75
5	RαMPAD (M5)	84.574	1.1

**Fig. 3:**
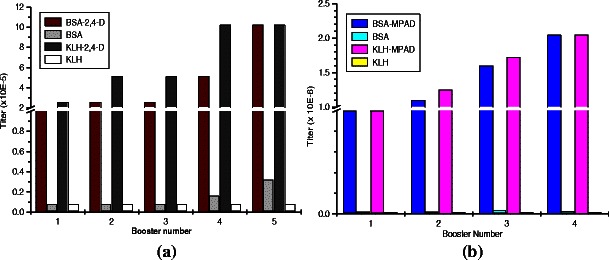
Antisera titer against: **a** BSA-2,4-D and **b** BSA-MPAD. Titer value was calculated where the O.D. was 0.1 and 1.0 for BSA-2,4-D and BSA-MPAD, respectively

By taking well-characterized hapten–protein conjugates, prepared at protein/hapten molar ratio of 1:40, significantly high titers of anti-2,4-D and anti-MPAD antibodies were observed (Fig. 3a & b). The titer of antibody was around 2 × 10^−5^ to 1 × 10^−6^ from third booster onward in case BSA-2,4-D immunized rabbits (Fig. 3a) and 5 × 10^−5^ to 2.0 × 10^−6^, with significantly low titer of anti-BSA antibodies after first booster onward in BSA-MPAD immunized rabbits (Fig. 3b). Antibodies were purified by protein A sepharose, and then using BSA-sepharose column to remove anti-carrier (BSA) antibodies. Antibody recovery with protein A sepharose-4B column was around 10–12 mg mL^−1^. Upon further purification by using sepharose-4B-BSA-column, the recovery was approximately 8 mg mL^−1^ of specific antibodies.

**Fig. 4:**
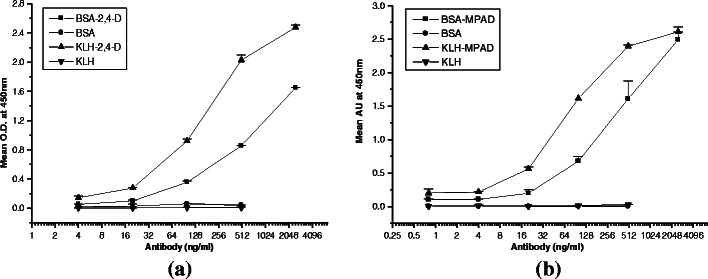
**a** Dilution curve for BSA column purified rabbit anti-BSA-2,4-D antibody against BSA-2,4-D, BSA, KLH-2,4-D, and KLH-coated wells in ELISA. **b** Dilution curve for BSA column purified rabbit anti-MPAD antibody against BSA-MPAD, BSA, KLH-MPAD, and KLH-coated wells in ELISA

**Fig. 5:**
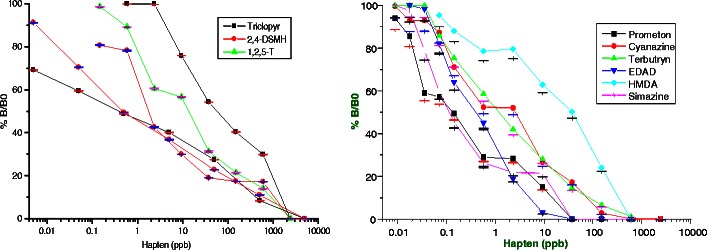
Cross-reactivities with different analogues of 2,4-D (**a**) and atrazine (**b**), respectively

BSA-2,4-D antibodies purified by protein A sepharose showed good reactivity against BSA-2,4-D and KLH-2,4-D conjugates, while showing almost negligible reactivity with carrier proteins BSA and KLH. Further purification by passing through the BSA–sepharose column, the antibody sensitivity was improved significantly, demonstrating the reactivity of the developed anti-2,4-D antibody approximately 6.25 ng mL^−1^ (Fig. 4a). Similarly, anti-BSA-MPAD antibody after protein A sepharose purification showed very high reactivity with conjugated MPAD and almost negligible reactivity with carrier protein BSA. The obtained reactivity for anti-atrazine antibody was app. 0.8 ng mL^−1^ (Fig. 4b). The percentage of cross-reactivity of different analogues of 2,4-D and atrazine are shown in Fig. 5a, b respectively. As can be seen in the figure, the antibody against 2,4-D and atrazine show a high degree of cross-reactivity with the tested analogues. This is along expected lines as it is a class specific antibody.

**Fig. 6:**
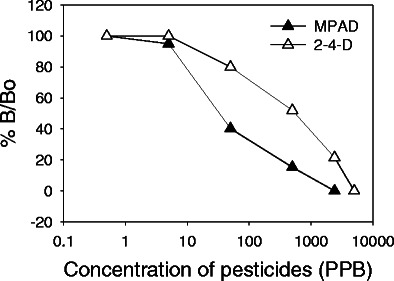
Competitive inhibition-based fluorescence immunoassay for atrazine and 2,4-D. The *curves* show the dilution curve analysis for analytes (atrazine and 2,4-D) concentrations between 0.5 to 5,000 ng mL^−1^. Free antigens were pre-incubated individually with respective antibody for 30 min before adding into microtiter plates

Competitive FIA was performed using direct hapten coated and also with plates coated with hapten–protein conjugates by using purified 2,4-D and atrazine antibodies at 1.6-μg mL^−1^ concentration each. For this, each antibody was pre-incubated with different concentrations of target molecules, 2,4-D and MPAD, and then checked for binding with respective conjugated hapten-coated plate and direct hapten-coated plates. The results show that for hapten–protein conjugate-coated plates, 50 % *B*/*B*_0_ signal was observed at 70 and 12 ng mL^−1^ of 2,4-D and atrazine concentration, respectively (Fig. 6)

In immunoassay-based pesticides detection, it is important to have the use of an antibody that demonstrates very high sensitivity as well as specificity. In many previous studies, polyclonal antisera as such have been used for estimating the levels of different pesticides [16]. However, only few groups have reported the use of purified antibodies for pesticides detection assay [17]. The present study demonstrates the efficient purification of antibodies with high yield using a combination of protein A sepharose column followed by passing over carrier protein column which resulted in total recovery of 90 % having around 75–80 % anti-hapten antibodies. The antibodies so obtained exhibited high sensitivity (Fig. 4a, b). The reactivity of purified antibody against specific hapten in conjugated hapten coated ELISA was 6.25 ng mL^−1^. The relative affinity constant of antibodies, as calculated with the computer program indicated that the anti-2,4-D and anti-MPAD antibody showed lower relative affinity by using conjugate coated plates 8.59 × 10^7^ and 9.28 × 10^8^ L mol^−1^.

An enzyme-linked immunosorbent assay for small molecules, in general, needs conjugates of the hapten with large carrier protein for coating the wells of microtiter ELISA plates. The formation of such conjugates is not always reproducible. This makes it difficult to evaluate hapten–protein stoichiometry and to understand the precise orientation of the hapten on the protein. Also, protein molecules while linked to hydrophobic polystyrene surface by passive adsorption might loose their activity and may suffer considerable denaturation. These macromolecules are found to better retain their functional activity when immobilized through extended hydrophilic spacer arms, since sorption on the surface is substantially reduced. In an ELISA, the sensitivity of the assay depends to a great extent on the degree of antigen binding to the microtiter plates. The binding of hapten to the microtiter plates was examined using the direct hapten-coated plates and by using hapten–protein conjugate on microtiter plates. The sensitivity of the assay obtained by using direct hapten coated plates was about 100-folds higher than the assay performed with hapten–protein conjugates with very high degree of reproducibility. The relative affinity demonstrated by using direct specific hapten coated plates 1.80 × 10^10^ and 1.9 × 10^10^ L mol^−1^ (detail curves are not depicted). This was mainly because of retention of functional activity of hapten molecules on polystyrene plates. Thus, after comparing the conjugated hapten-coated and direct hapten-coated plate for 2,4-D and atrazine detection, it was observed that the sensitivity of antibody in direct hapten-coated format was significantly improved. No lose of functional activity of hapten molecules which is an organic moiety was observed, as reported in case of biomolecular immobilization on polystyrene plates.
